# LAT1 (SLC7A5) and CD98hc (SLC3A2) complex dynamics revealed by single-particle cryo-EM

**DOI:** 10.1107/S2059798319009094

**Published:** 2019-06-28

**Authors:** George N. Chiduza, Rachel M. Johnson, Gareth S. A. Wright, Svetlana V. Antonyuk, Stephen P. Muench, S. Samar Hasnain

**Affiliations:** a Molecular Biophysics Group, Institute of Integrative Biology, Faculty of Health and Life Sciences, University of Liverpool, Liverpool L69 7ZB, England; b School of Biomedical Sciences and Astbury Centre for Structural Molecular Biology, University of Leeds, Leeds LS2 9JT, England

**Keywords:** transporters, cryo-EM, amino-acid transporters, solute carriers, LAT1, CD98hc, SLC7A5, SLC3A2

## Abstract

Solute carriers are a large class of transporters that play key roles in normal and disease physiology. Among the solute carriers, heteromeric amino-acid transporters (HATs) are unique in their quaternary structure. LAT1–CD98hc, a HAT, transports essential amino acids and drugs across the blood–brain barrier and into cancer cells. It is therefore an important target both biologically and therapeutically. During the course of this work, cryo-EM structures of LAT1–CD98hc in the inward-facing conformation and in either the substrate-bound or apo states were reported to 3.3–3.5 Å resolution [Yan *et al.* (2019), *Nature (London)*, **568**, 127–130]. Here, these structures are analyzed together with our lower resolution cryo-EM structure, and multibody 3D auto-refinement against single-particle cryo-EM data was used to characterize the dynamics of the interaction of CD98hc and LAT1. It is shown that the CD98hc ectodomain and the LAT1 extracellular surface share no substantial interface. This allows the CD98hc ectodomain to have a high degree of movement within the extracellular space. The functional implications of these aspects are discussed together with the structure determination.

## Abstract 2

Insight is provided into the dynamic interactions of LAT1 and CD98hc and their role in the transport cycle of the complex.

## 1. Introduction   

Solute carriers (SLCs) are an important class of membrane proteins that are involved in the transport of nutrients, signalling molecules and various metabolites as well as drugs and their catabolites. They play key roles in human health and disease and are important for normal and aberrant physiology. Compared with other gene families of similar size and physiological relevance, the function and molecular mechanisms of SLC transporters are poorly understood, in part owing to a lack of structural information (César-Razquin *et al.*, 2015 ▸; Bai *et al.*, 2017 ▸). Heterodimeric amino-acid transporters (HATs) are rare among the estimated 400 SLC transporter genes annotated in the human genome (Schlessinger *et al.*, 2010 ▸). There are seven HATs with subunits belonging to the *slc*3 and *slc*7 gene families, each with different functionality. SLC3A1 and SLC3A2 are the heavy chains of the HATs, which act as chaperones for the translocation of SLC7-family transport-active light chains to the plasma membrane. SLC3A2, also known as CD98hc or 4F2hc, forms heterodimers with SLC7A5–SLC7A8 and SLC7A10–SLC7A11, while SLC3A1 complexes with SLC7A9 (Fotiadis *et al.*, 2013 ▸; Devés & Boyd, 2000 ▸). The two HAT subunits are linked together by a disulfide bridge between a conserved cysteine in the loop between transmembrane helices 3 and 4 of the light chain and a conserved cysteine in the heavy chain (Fotiadis *et al.*, 2013 ▸; Devés & Boyd, 2000 ▸).

The L-type amino-acid transporter 1/SLC7A5 (LAT1) is a 55 kDa polytopic integral membrane protein that has been shown to function as an Na^+^-independent secondary active antiporter of neutral l-amino acids and in some cases their catabolites. Substrates of LAT1 include leucine, isoleucine, valine, phenylalanine, tyrosine, tryptophan, methionine and histidine (Mastroberardino *et al.*, 1998 ▸; Kanai *et al.*, 1998 ▸). CD98hc is a 68 kDa type II glycoprotein that functions as a chaperone for LAT1, stabilizing and facilitating its translocation to the plasma membrane (Nakamura *et al.*, 1999 ▸). LAT1 is expressed in a number of tissues throughout the body (in descending magnitude of expression: foetal liver, placenta, brain, testis, bone marrow and leucocytes), whereas CD98hc has been reported to be expressed more ubiquitously (Yanagida *et al.*, 2001 ▸). The expression of LAT1–CD98hc in placenta has been hypothesized to be essential for foetal development owing to its role in the uptake of essential amino acids and thyroid hormones (Ritchie & Taylor, 2001 ▸). Moreover, an increasing number of tumours have been shown to ectopically express the LAT1–CD98hc complex (Zhao *et al.*, 2015 ▸; Cantor & Ginsberg, 2012 ▸). It has been hypothesized that CD98hc plays a similar role in cancer cells as in lymphocyte activation, which is the amplification of β1 and β3 integrin signalling, reducing anchorage dependence and promoting cell proliferation by modulating cyclin-dependent kinase regulation through ERK signalling (Cantor & Ginsberg, 2012 ▸). Moreover, the transport activity of LAT1–CD98hc has been shown to be pro-tumorigenic (Napolitano *et al.*, 2015 ▸). LAT1 substrates are necessary for protein synthesis and for the replenishment of the tricarboxylic acid cycle intermediates that are used in the synthesis of other macromolecules such as nucleotides, as is required for the survival and dysregulated proliferation of tumour cells (DeBerardinis *et al.*, 2007 ▸). Leucine, which is one of the substrates of LAT1, is sensed by sestrin2, leading to the activation of mTORC1, which in turn promotes cell growth while inhibiting autophagy (Saxton *et al.*, 2016 ▸; Nicklin *et al.*, 2009 ▸; Walls *et al.*, 2016 ▸). LAT1 has also been shown to transport drugs such as l-DOPA and gabapentin across the blood–brain barrier (BBB; Dickens *et al.*, 2013 ▸; Kageyama *et al.*, 2000 ▸). It is estimated that only 2% of small-molecule drugs can cross the BBB. Central nervous system penetrance therefore poses a significant hurdle for the development of small-molecule therapeutics for neurological diseases (Pardridge, 2005 ▸). LAT1–CD98hc is thus an important drug target for chemotherapy and drug delivery.

Structures of LAT1–CD98hc in the inward-facing conformation at 3.3 and 3.5 Å resolution have recently been reported,^1^ revealing extensive interaction between the transmembrane and intracellular domains of CD98hc and LAT1, with limited interaction between the ectodomain and LAT1. The transmembrane interaction region is mediated by direct hydrophobic protein–protein contacts along the length of the helices and indirectly by lipids. Yan *et al.* (2019 ▸) and Lee *et al.* (2019 ▸) proposed that polar interactions between the extracellular domain of CD98hc and the extracellular surface of LAT1 would have important consequences for transport. Similarly, Rosell and coworkers combined *in silico* docking of the CD98hc crystal structure and a homology model of LAT2 with mutagenesis and cross-linking experiments and proposed an extensive dimer interface, with the CD98hc ectodomain covering ∼1735 Å^2^ of the extracellular face of LAT2. This extensive interaction was suggested to be the mechanism by which CD98hc stabilizes LAT2 and has been assumed to be the same for other light chains such as LAT1 (Rosell *et al.*, 2014 ▸; Dickens *et al.*, 2017 ▸).

We have previously reported the kinetic and thermodynamic stabilization of detergent-solubilized LAT1–CD98hc by cholesterol hemisuccinate, which has proven to be important for cryo-EM studies (Newstead, 2019 ▸). Here, we report on the dynamics of LAT1–CD98hc observed by single-particle cryo-EM, revealing flexibility in the interaction between the two subunits on the extracellular side.

## 2. Methods   

### 2.1. Purification   

LAT1–CD98hc was expressed and purified as reported previously and immunoblotting was performed in the same manner (Dickens *et al.*, 2017 ▸). Briefly, HEK293 suspension-adapted and GNTI^−^ (HEK293SG) cells stably overexpressing V5 epitope-tagged LAT1 were lysed by sonication in Dulbecco’s phosphate-buffered saline pH 7 with 1 m*M* sodium aurothiomalate. Crude membranes were prepared from the cells by ultracentrifugation and were solubilized overnight with anti-V5 resin in 20 m*M* Tris–HCl, 300 m*M* NaCl, 10% glycerol supplemented with 0.9%(*w*/*v*) *n*-dodecyl β-maltoside (DDM), 0.19%(*w*/*v*) cholesteryl hemisuccinate Tris salt (CHS) and 0.1%(*w*/*v*) lauryl maltose neopentyl glycol (LMNG). The HAT complex was eluted from the resin using V5 peptide and was then applied onto a Superdex 200 10/300 column in 100 m*M* Tris–HCl, 300 m*M* NaCl supplemented with 0.01% DDM/CHS/LMNG in a 15:3:1 ratio before cryo-EM.

### 2.2. Grid preparation and data collection   

Cryo-grids were prepared at 2.3 mg ml^−1^. 3 µl aliquots were applied onto glow-discharged Quantifoil Au R1.2/1.3 holey carbon grids. The grids were plunge-frozen in liquid ethane using a Vitrobot Mark IV (FEI), with grids blotted for 6 s at a blot force of 6 and maintained at 100% humidity and 4°C. Data were collected using a Titan Krios microscope at eBIC (Diamond Light Source) operating at 300 kV equipped with a K2 Summit detector (Gatan) with the Volta phase plate inserted. Automated data collection was performed with the *EPU* software at a magnification of 47 710×, using a defocus value of −0.7 µm. A total of 2390 micrographs were collected with a pixel size of 1.06 Å. The total dose, 51 e^−^ Å^−2^, was acquired using a dose rate of 4.96 e^−^ per pixel per second across 40 frames for 12 s total exposure. Several data sets were collected, at the University of Leeds Biostructure EM facility, with and without the phase plate and in different buffer conditions. Little difference was observed in the resultant reconstructions and the best data set is reported and discussed below. Data-collection parameters are given in Table 1 ▸.

### 2.3. Cryo-EM data processing   

Whole-frame beam-induced motion correction and CTF correction were performed using *MotionCorr*2 and *Gctf* (v.1.06), respectively (Zheng *et al.*, 2017 ▸; Zhang, 2016 ▸). Using a box size of 200 Å, 1388 particles were manually picked in *RELION*-3 (Zivanov *et al.*, 2018 ▸) and subjected to reference-free 2D classification to yield 2D classes for use as auto-picking references. The 2D references were used to auto-pick 374 941 particles, giving an average of 157 particles per micrograph. Using a previously generated initial 3D model low-pass filtered to 60 Å, 3D classification using all of the particles was performed with two classes in order to remove contaminants and false positives from auto-picking, while reducing the risk of discarding poorly represented orientations of the HAT. After visual inspection, 191 445 particles that were in one of the two classes were chosen and subjected to reference-free 2D classification to yield 100 classes, the majority of which were the protein–detergent complex. After 2D classification of this particle set and 3D classification of the particles making up the best 2D classes, 77 423 particles remained and were used in 3D auto-refinement, yielding a 3D single-particle reconstruction at 12 Å resolution after post-processing and a *B*-factor sharpening of −1200 Å^2^.

### 2.4. Interpretation of Coulomb potential maps produced by single-particle reconstruction   

Docking of the apo LAT1–CD98hc structure (PDB entry 6irs; Yan *et al.*, 2019 ▸) was performed using the ‘Fit in map’ tool in *UCSF Chimera*. The docking of the crystal structure of the ectodomain of CD98hc (PDB entry 2dh2; Fort *et al.*, 2007 ▸) into the EM maps, after filtering the crystal structure to 12 Å resolution, was performed by segmenting the map using the *SEGGER* (v.1.9.4) tool with the map at a threshold of 0.00872; the ‘Smoothing Steps’ parameter in *SEGGER* was set to ten steps, while the other options were set to the default values (Pintilie *et al.*, 2010 ▸). An apo-out open model of LAT1 was docked into the map after segmenting at the same threshold but with ‘Smoothing Steps’ set to 3. Several segments were grouped to form a single segment that contained the density at the centre of the larger lobe of the map. The model was fitted after filtering to 12 Å.

### 2.5. Multibody 3D auto-refinement   

A two-body multibody refinement was performed as a continuation of the final 3D auto-refinement. The density corresponding to the ectodomain of CD98hc was defined as body 1 and the micellar density as body 2. Density maps for mask creation were created by segmenting the 12 Å resolution map using *SEGGER* into two densities corresponding to each body to be refined; the map segments were Gaussian-filtered using the ‘Volume Filter’ tool and resampled using the vop command onto the same grid as the 12 Å resolution map using *UCSF Chimera*. These map segments were then low-pass filtered with 30 Å and 11 Å soft edges added in *RELION*-3 to create the masks used in the refinement. The body.star file was set up as described by Nakane *et al.* (2018 ▸). Principal component analysis was performed on the orientations of both bodies, and movies for the first three principal components (PCs) were written out as a series of volumes in MRC format (Crowther *et al.*, 1996 ▸) describing the relative motion of the two bodies as described by these PCs. The ‘Volume series’ tool of *UCSF Chimera* was used to visualize the movies and the ‘Fit in map’ tool was used to fit CD98hc ectodomain and the transmembrane domains of LAT1–CD98hc from the structure with PDB code 6irs or the homology model of LAT1 in each of the volumes before saving each as a PDB file to be used for ensemble analysis using the ‘MD movie’ tool in *UCSF Chimera*.

### 2.6. Modelling LAT1 and docking of the CD98hc ectodomain   

The sequence of LAT1 (NCBI accession No. NP_003477.4) was submitted to the *HHpred* server (Söding *et al.*, 2005 ▸) to search for homology-modelling templates in the Protein Data Bank using default parameters. The apo outward-facing structure of AdiC from *Escherichia coli* (PDB entry 5j4i; Ilgü *et al.*, 2016 ▸) was chosen as a template for modelling LAT1 using *MODELLER* v.9.19 (Webb & Sali, 2016 ▸). Ten decoys were generated and the decoy with the lowest DOPE (discrete optimized protein energy) score was carried forward for optimization via *ModRefiner* using a template (Xu & Zhang, 2011 ▸). The CD98hc ectodomain (PDB entry 2dh2) and LAT1 were docked using *ClusPro* (Kozakov *et al.*, 2017 ▸). Distance restraints were provided based on LAT2 cross-linking experiments and the conserved inter-subunit disulfide bond (Supplementary Fig. S1). The LAT1 sequence was also submitted to *ConSurf* (Ashkenazy *et al.*, 2016 ▸) for conservation analysis using default settings.

## 3. Results   

### 3.1. Struture and inter-domain dynamics of LAT1–CD98hc   

**Figure 1:**
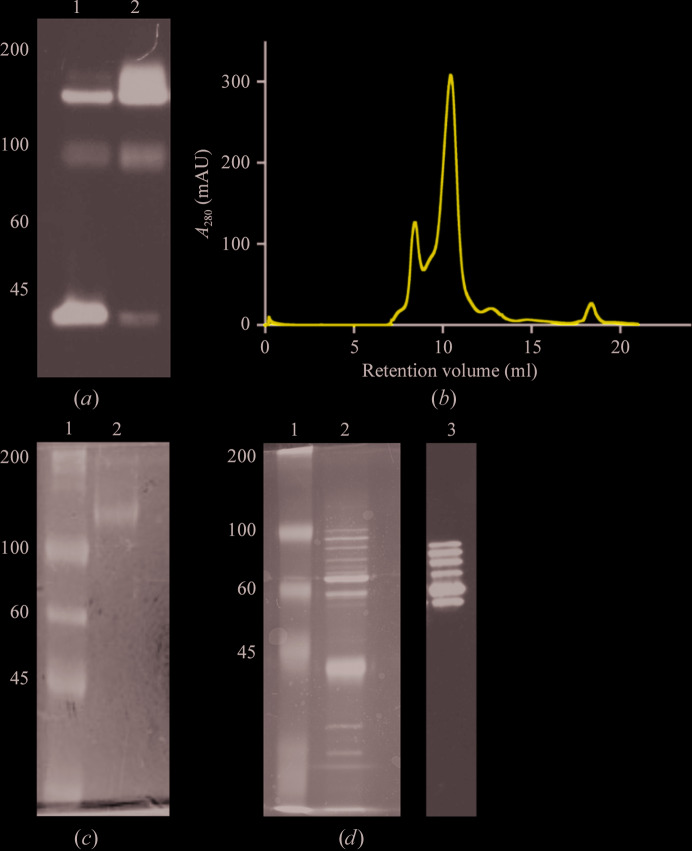
Purification of LAT1–CD98hc. (*a*) Immunoblot using anti-LAT1 polyclonal antibody under nonreducing conditions of HEK293 cells overexpressing LAT1 that were lysed in the absence (lane 1) or presence (lane 2) of 1 m*M* sodium aurothiomalate. The two bands that are visible are consistent with heterodimeric (123 kDa) and monomeric (55 kDa) LAT1. (*b*) Size-exclusion chromatogram of LAT1–CD98hc after purification by immunoprecipitation on a Superdex 200 10/300 column at 0.4 ml min^−1^. The peak at a retention volume of 10.5 ml was used for cryo-EM. (*c*) Coomassie-stained SDS–PAGE gel of purified LAT1–CD98hc (lane 2) run under nonreducing conditions. (*d*) Coomassie-stained SDS–PAGE gel of purified LAT1–CD98hc (lane 2) run under reducing conditions and immunoblotted using anti-CD98hc monoclonal antibody (lane 3). Molecular-weight markers are labelled on the left in kDa.

LAT1 was stably expressed in HEK293SG cells and endogenous CD98hc was upregulated in response, as reported previously (Khunweeraphong *et al.*, 2012 ▸; Dickens *et al.*, 2017 ▸). Sodium aurothiomalate was added to the lysate to prevent reduction of the conserved inter-subunit disulfide bond, and immunoaffinity was used to purify the heteromeric complex after detergent solubilization [Figs. 1 ▸(*a*), 1 ▸(*b*) and 1 ▸(*c*)]. Size-exclusion chromatography yielded a major peak eluting at 10.5 ml, which was determined to be LAT1–CD98hc with an intact inter-subunit disulfide bond. This product was of sufficient purity for structural characterization by cryo-EM [Figs. 1 ▸(*b*), 1 ▸(*c*) and 1 ▸(*d*)].

**Figure 2:**
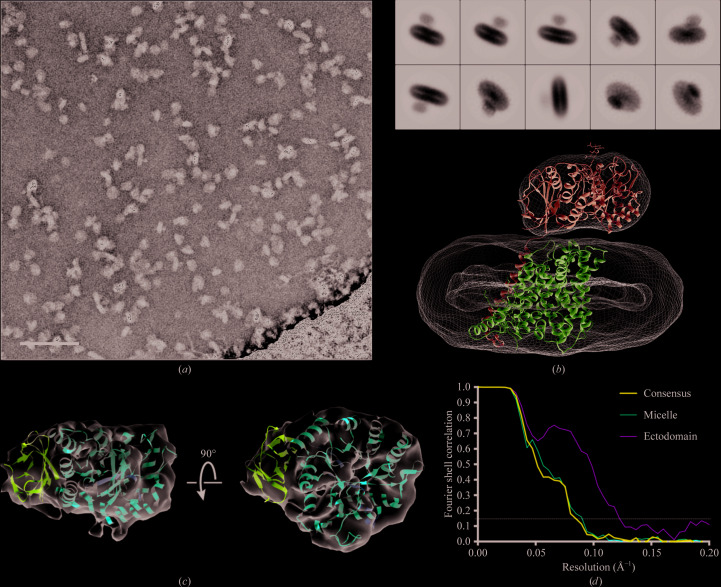
Cryo-EM and single-particle reconstruction of detergent-solubilized LAT1–CD98hc. (*a*) Micrograph of LAT1–CD98hc collected with the Volta phase plate after motion and CTF correction. The scale bar shown in the bottom left corner is 60 nm in length. The particles observed are consistent in size and shape with a heterodimeric, 123 kDa complex in DDM/CHS. (*b*) Representative 2D classes of the particles of LAT1–CD98hc used in 3D reconstruction; below, the resulting 3D map. The 2D classes and 3D map (mesh) are congruent and are characteristic of a HAT protein–detergent complex, with a small extracellular density and a large ellipsoidal detergent belt. LAT1 (magenta) and CD98hc (blue; PDB entry 6irs) are shown as ribbon models docked into the 3D map (EMD-4642). The particle box size was 200 Å with a mask diameter of 180 Å. (*c*) EM map density corresponding to the CD98hc ectodomain after multibody refinement with the crystal structure docked (PDB entry 2dh2). The A and C domains of CD98hc are coloured purple and red, respectively. (*d*) Fourier shell correlation (FSC) curves for the consensus reconstruction and for the ectodomain and micelle reconstructions. The resolution at a 0.143 FSC cutoff is ∼12 Å (consensus, blue), 11 Å (micelle, red) and 9 Å (ectodomain, green).

Single particles visible in the micrographs were consistent with a homogeneous sample of a 123 kDa protein–detergent complex and showed a clear bilobed structure [Fig. 2 ▸(*a*)]. 2D classification revealed an asymmetric bilobed structure [Fig. 2 ▸(*b*)] similar to those observed in the recently reported cryo-EM structures of the LAT1–CD98hc complex in similar detergent conditions [Fig. 2 ▸(*b*)] (Yan *et al.*, 2019 ▸). The larger lobe had an ellipsoidal geometry characteristic of the *n*-dodecyl maltoside and cholesterol hemisuccinate micelles (DDM/CHS) used here to purify and stabilize LAT1–CD98hc (O’Malley *et al.*, 2011 ▸; Dickens *et al.*, 2017 ▸). 3D reconstruction from this data set yielded an ∼12 Å resolution map of the transporter complex as determined by gold-standard Fourier shell correlation (FSC; 0.143 cutoff) [Fig. 2 ▸(*d*)]. One of the high-resolution strutures of LAT1–CD98hc (PDB entry 6irs) was docked into the map, with the ectodomain of CD98hc occupying the smaller lobe and the transmembrane regions of the two subunits occupying the detergent micelle density [Fig. 2 ▸(*b*)]. The crystal structure of the CD98hc ectodomain (PDB entry 2dh2) was docked into the smaller lobe of the EM envelope after two-multibody 3D auto-refinement as described below. The depression at the centre of the triosephosphate isomerase-like barrel in the A domain of CD98hc permitted orientation of the crystal structure in the EM map accurately [Fig. 2 ▸(*c*)]. The larger lobe of the 3D reconstruction had a ring of missing density which may represent the boundary between the detergent micelle and the transmembrane domains of LAT1 and CD98hc [Fig. 2 ▸(*b*)]. There was no density between the two lobes and most of the backbone of the docked structures was accounted for by the density, indicating the absence of extensive interaction between the two subunits on the extracellular side [Fig. 2 ▸(*b*)].

**Figure 3:**
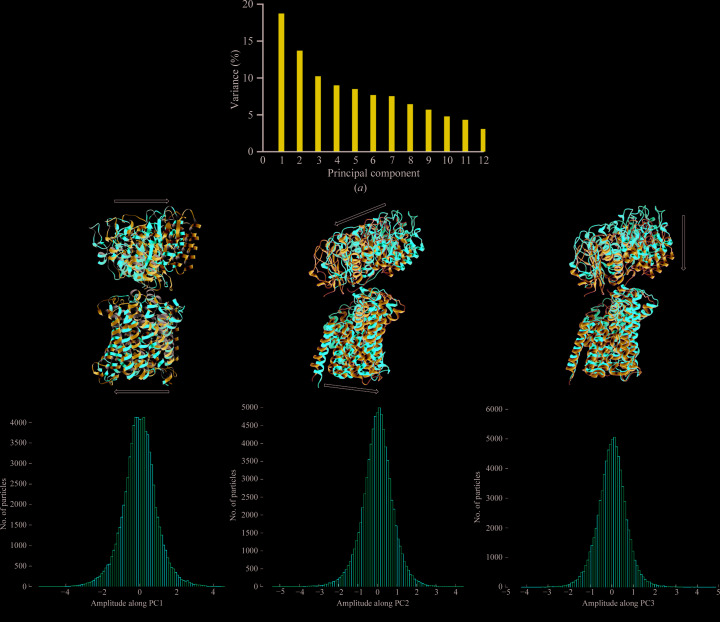
Two-body 3D auto-refinement and principal component analysis of their rotations and translations. (*a*) Variance in the data set was captured in 12 principal components. The first three components accounted for 42.7% of the variance, and volume series along these components were interpreted by docking the CD98hc ectodomain and LAT1. Motion along the first (*b*), second (*c*) and third (*d*) principal components (upper panel) and the histograms of the amplitudes (lower panel) along them are shown. The first and last frames of the volume series along each vector are coloured red and blue, respectively.

To explore the flexibility between the two lobes of the LAT1–CD98hc structure, 3D multibody auto-refinement was performed. This was followed by principal component analysis (PCA) as described by Nakane *et al.* (2018 ▸). PCA of the variance in rotations and translations of the two bodies defined in the multibody refinement was accounted for in 12 principal components (PCs), with the first three accounting for 42.7% of the variance [Fig. 3 ▸(*a*)]. The distribution of variance across the PCs shows a limited preference for variance along a particular PC, suggesting almost independent movement of the two lobes (Lever *et al.*, 2017 ▸). In order to visualize the molecular flexibility between the two bodies, a volume series consisting of ten volumes per component was rendered out for the first three components. The crystal structure of the CD98hc ectodomain and the structure of the transmembrane domains of the complex were docked into each volume of the three series, allowing modelling of the flexibility described by their principal components in molecular terms. Component 1, which described 18.7% of the variance, showed mostly translation but also some rotation of the CD98hc ectodomain and LAT1 in opposite directions along parallel planes perpendicular to the plane of the long axis of the complex [Fig. 3 ▸(*b*); Supplementary Movie S1]. The second largest principal component, which described 13.7% of the variance, showed a forward, downward and slanted motion in the CD98hc ectodomain and a downward backward motion in LAT1, with the effect of bringing the C domain of the CD98hc ectodomain closer to LAT1 while lifting the A domain away in the final frame [Fig. 3 ▸(*c*); Supplementary Movie S2]. The third component showed upward and downward motions in the CD98hc ectodomain and a slight twisting motion in LAT1, bringing the two closer to each other and then further apart [Fig. 3 ▸(*d*); Supplementary Movie S3]. The unimodal distribution of particles along these principal components suggests that the motion along these principal components is continuous [Figs. 3 ▸(*b*), 3 ▸(*c*) and 3 ▸(*d*)].

### 3.2. The role of intersubunit interaction in the transport mechanism   

**Figure 4:**
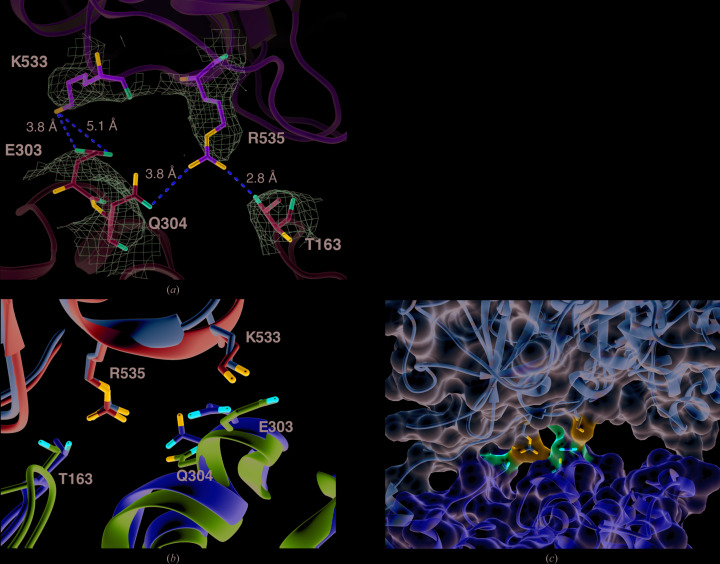
Interface of LAT1 and the CD98hc ectodomain. (*a*) Residues proposed to be involved in electrostatic interactions between the two subunits on the extracellular side. CD98hc is shown in green and LAT1 is in cyan (PDB entry 6irt). The EM density map on which the model is based is shown as a mesh (EMD-9722). (*b*) Side-chain conformations of these putatively interacting residues from the substrate-bound and inhibitor-incubated strutures of LAT1–CD98hc (PDB entries 6irt and 6irs, respectively). (*c*) Surface of the extracellular interface of substrate-bound LAT1–CD98hc, highlighting the solvent-accessibility of the interface. Putative interacting residues are showns as sticks.

In addition to the intersubunit disulfide bond, electrostatic interactions between residues on the extracellular side of LAT1 and the CD98hc ectodomain have been proposed to stabilize the interaction between the two subunits (Yan *et al.*, 2019 ▸). However, there is no EM density in the maps of the 3.3 and 3.5 Å resolution structures corresponding to some of the positions of the side chains of these putative interacting residues, and this may perhaps be why these residues have different conformations in the two structures. This is particulary striking for Gln304 and Glu303 in LAT1, which are modelled on a helix for which there is no corresponding EM density (PDB entry 6irs) [Figs. 4 ▸(*a*) and 4 ▸(*b*)]. The lack of density in these regions of the map suggests flexibility in these residues, especially in the case of the helix bearing Gln304 and Glu303 in LAT1. The average distances between the putative interacting pairs, 3.5 Å from Arg535 (CD98hc) to Thr163 (LAT1), 5 Å from Arg535 to Gln304 and 4.4 Å from Lys533 to Glu303, as well as the potentially attenuating effect of the solvent accessibility, are both consistent with a weak stabilizing interaction, flexibility in these residues and thus dynamic interaction between LAT1 and the CD98hc ecto­domain [Fig. 4 ▸(*c*)].

**Figure 5:**
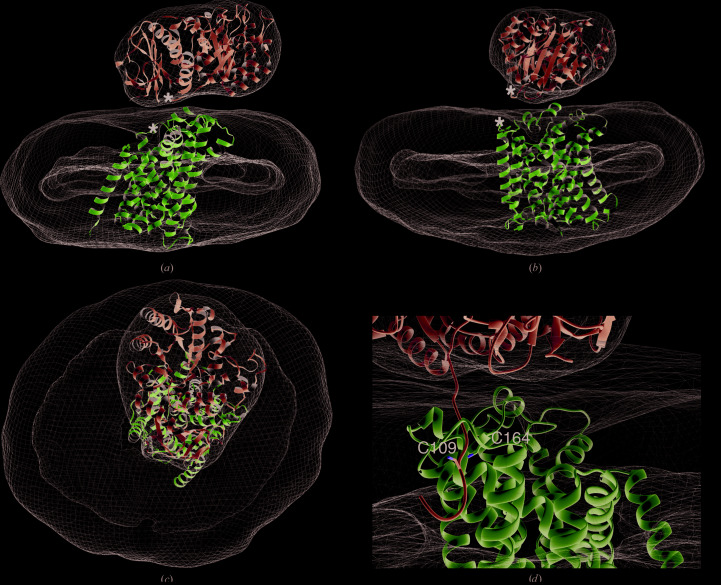
Docking of LAT1 in the outward open conformation into a 12 Å resolution EM map. Side (*a*), front (*b*) and top (*c*) views of the docked EM map, represented as a mesh with the CD98hc ectodomain crystal structure shown in blue and a homology model of LAT1 in pink, are shown. Asterisks mark the positions of Gly109 in CD98hc and Cys164 in LAT1. (*d*) The interdomain linker, Gly109–Gly127, of CD98hc modelled to show the plausibility of inter-subunit disulfide-bond formation between Cys164 of LAT1 and Cys109 of CD98hc (shown as sticks with S atoms in yellow) docked into the EM map (shown as a mesh).

To explore whether the dynamics of LAT1–CD98hc interaction were particular to the inward-facing conformation common to the high-resolution structures reported to date, homology modelling was used to predict the structure of the outward open conformation. Despite the low resolution of the EM map, utilizing our knowledge of the extracellular local­ization of the CD98hc crystal structure and the location of the inter-subunit disulfide bond, as well as the orientation of LAT1 in the membrane, we were able to dock a homology model of LAT1 in the outward open conformation into the map (Fig. 5 ▸). The distance between Cys164 in the outward open model of LAT1 and Gly109, which substituted for Cys109 in the CD98hc ectodomain structure, was found to be ∼15 Å in the EM-derived model [Figs. 5 ▸(*a*), 5 ▸(*b*) and 5 ▸(*c*)]. Various conformations of the loop were modelled to demonstrate the plausibility of disulfide bonding between the HAT subunits as docked in the EM map [Fig. 5 ▸(*d*)]. The volume series showing the flexibility of the interaction between the CD98hc ectodomain and LAT1 was similarly interpretable using the homology model as with the high-resolution structure, indicating that the dynamics are not specific to either the outward open or inward open conformations of LAT1. This suggests that the dynamics of the extracellular side of the complex do not play a role in the conformational changes of LAT1 during its transport cycle.

**Figure 6:**
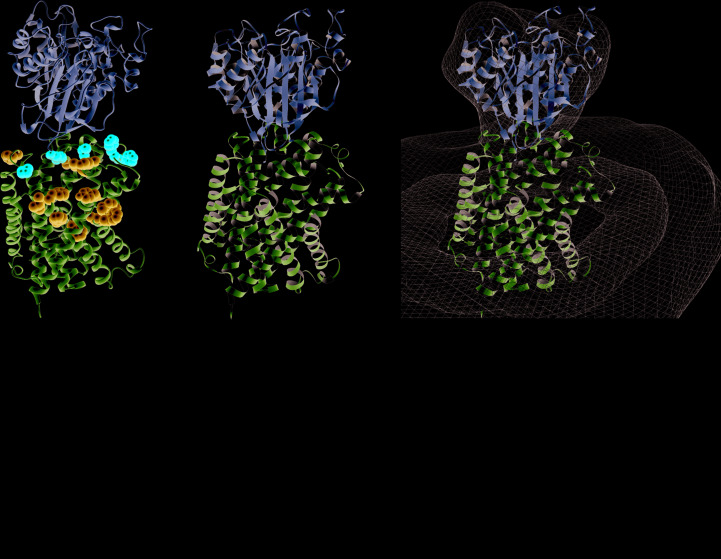
Comparison of the LAT1–CD98hc dimer interface predicted from homology to LAT2 with EM data. (*a*) Model of the CD98hc ectodomain (tan) and LAT1 (pink) from docking into the EM map shown as a ribbon with putative interfacial residues shown as spheres. Residues within 10 Å of the CD98hc ectodomain in the EM-based model are shown in red and those at >10 Å are shown in blue (Supplementary Table S2). (*b*) The docking solution of the CD98hc ectodomain and LAT1 shows a similar orientation of subunits as in the consensus cryo-EM map. (*c*) An *in silico* heterodimer model docked into the last frame of the volume series of PC3. (*d*) The distance between the N atoms of residues of LAT1 (chain A) and CD98hc (chain B) across the frames of a molecular-dynamics movie generated from the volume series along the first three principal components describing the variance between LAT1 and CD98hc in the EM data. The legend gives the residue name, chain and atom.

It has been suggested that the ectodomain of CD98hc interacts with the plasma membrane. Our analysis and the high-resolution structures are based on detergent-solubilized LAT1–CD98hc and therefore do not address the role of this membrane interaction in the structure and function of the complex (Fort *et al.*, 2007 ▸). The extracellular face of LAT2 and the ectodomain of CD98hc have been postulated to interact extensively, with a predicted interface of ∼1735 Å^2^, based on cross-linking experiments performed in the native environment of the cell plasma membrane (Rosell *et al.*, 2014 ▸). In all of the reported structures of LAT1, LAT1 and the ectodomain of CD98hc do not interact in the manner suggested for LAT2 (Figs. 2 ▸ and 3 ▸). Even though there are similar residues to those predicted to be present at the interface of the CD98hc ectodomain and LAT2 on the LAT1 extracellular face, some of these residues are not conserved across LAT1 orthologues, suggesting that they may not play significant structural and or functional roles [Fig. 6 ▸(*a*); Supplementary Table S2]. Docking of the CD98hc ectodomain and LAT1 was attempted but failed to give solutions that satisfied distance restraints derived from the cross-linking of LAT2 and CD98hc in the plasma membrane [Figs. 6 ▸(*b*) and 6 ▸(*c*)]. The distance between residues that were positive in LAT2–CD98hc cross-linking experiments were tracked across each frame of the volume series for PC1–PC3. All residues tracked were >18 Å apart in all 30 frames of the three major principal components [Fig. 6 ▸(*d*); Supplementary Table S2]. This was outside the range of the cross-linkers used by Rosell *et al.* (2014 ▸) (3.5–14.3 Å; Supplementary Table S2).

## 4. Discussion   

Heterodimeric transporters are rare among SLC transporters, and the structural and functional significance of this unique quaternary structure remains to be thoroughly explored. We report here the dynamical interaction of the ectodomain of CD98hc and LAT1 as revealed by single-particle cryo-EM. Two-body 3D auto-refinement, followed by principal component analysis (PCA) of the rotations and translations of densities corresponding to the transmembrane domains/detergent micelle and CD98hc ectodomain, revealed variance within the 2D projections of the protein–detergent complex. The flexibility described in the first three principal components suggested that the CD98hc ectodomain does not interact with LAT1 and is only tethered to LAT1 through interactions of its transmembrane domain with those of LAT1 and by the conserved inter-subunit disulfide bond. The hypothesis that a weak noncovalent interaction exists between the heavy and light chain in a HAT was first put forward by Pfeiffer *et al.* (1998 ▸) based on the results of mutagenesis and immuno­precipitation experiments as well as the observation that the disulfide bond between the subunits is highly conserved. K533E and E303K mutations in CD98hc have previously been shown to reduce transport, which was interpreted as indicating the importance of putative interactions on the extracellular side of the complex to function (Yan *et al.*, 2019 ▸). *In silico* mutagenesis of these residues indicates that they are likely to create steric clashes across the extracellular interface.

We suggest, based on the analysis above, that the limited interaction of the CD98hc ectodomain and LAT1 is independent of the conformation of LAT1, at least with respect to the open inward-facing and outward-facing conformations. This is consistent with the flexibility of the linker between the transmembrane domain and the ectodomain of CD98hc and of the loop region between transmembrane helices 3 and 4 of LAT1, on which the conserved intersubunit disulfide bond is located. Flexibility in this region may also allow some degree of independence between the conformational dynamics of the transmembrane domains, which are essential for the transport cycle, and those of the ectodomain of the complex, as discussed here. The solution of high-resolution strutures of the complex in conformations other than the open inward-facing would test this hypothesis, allowing further exploration of the role of CD98hc in the transport mechanism of the complex.

An interaction interface between the CD98hc ectodomain and LAT2 was proposed on the basis of docking and cross-linking experiments by Rosell *et al.* (2014 ▸). LAT2 shares 52% sequence identity with LAT1 and the residues at this interface are similar; therefore, it had been assumed that LAT1 would interact with the CD98hc ectodomain in a similar manner. Our data suggest a more flexible interaction between the two subunits. Our analysis is consistent with the recently reported 13 Å resolution cryo-EM structure of detergent-solubilized LAT2–CD98hc and the recent ∼3 Å resolution structures of LAT1–CD98hc (Jeckelmann & Fotiadis, 2019 ▸; Yan *et al.*, 2019 ▸; Lee *et al.*, 2019 ▸). The cross-linking data on LAT2–CD98hc were derived from a complex that was cross-linked in the native environment of the plasma membrane. This may explain some of the discrepancy between the cross-linking data and the structure of the detergent-solubilized complexes (Rosell *et al.*, 2014 ▸), with detergent having been shown to impact the interactions and dynamics of membrane proteins (Chipot *et al.*, 2018 ▸; Seddon *et al.*, 2004 ▸). Therefore, an important next step is studying the LAT1 structure and its interactions in a more native lipid environment. We found no evidence of dimers of heterodimers, as has been suggested in the literature (Napolitano *et al.*, 2017 ▸). Fort *et al.* (2007 ▸) put forward a model for CD98hc homodimerization and plasma-membrane interaction based on the crystal structures of the ectodomain; if this model is correct then light chains such as LAT1 perhaps disrupt or prevent the formation of CD98hc homodimers (Fort *et al.*, 2007 ▸). Given the variety of contexts in which CD98hc is expressed, studying its oligomeric state in each context and its impact on the function of the protein may produce insights into the mechanisms of this glycoprotein.

## Supplementary Material

Supplementary Tables, Supplementary Figure and captions for Supplementary Movies. DOI: 10.1107/S2059798319009094/qh5062sup1.pdf

Visualization of conformational dynamics of CD98hc ectodomain (tan) and LAT1 (pink),along PC1. DOI: 10.1107/S2059798319009094/qh5062sup2.mp4

Visualization of conformational dynamics of CD98hc ectodomain (tan) and LAT1 (pink) along PC2. DOI: 10.1107/S2059798319009094/qh5062sup3.mp4

Visualization of conformational dynamics of CD98hc ectodomain (tan) and LAT1 (pink) along PC3. DOI: 10.1107/S2059798319009094/qh5062sup4.mp4

## Figures and Tables

**Table 1:** Data-collection parameters

Microscope and detector	Titan Krios with K2 Summit (Gatan)
Voltage (kV)	300
Phase plate	Yes
Pixel size (Å)	1.06
Defocus (µm)	−0.7
Total dose (e^−^ Å^−2^)	51
No. of frames	40
Dose per frame (e^−^ Å^−2^)	1.27
No. of micrographs	2390
Total auto-picked particles	374941
Particles in final refinement	77423

## Footnotes

1 These structures appeared when our original manuscript was under consideration by *Nature Communications* (submitted 4 March 2019). The current article has been rewritten in view of the paper by Yan *et al.* (2019 ▸).
