# Supplementary material for: LAT1 (SLC7A5) and CD98hc (SLC3A2) complex dynamics revealed by single-particle cryo-EM
Source: Acta Crystallogr D Struct Biol. 2019 Jun 28;75(Pt 7):660–9. doi: 10.1107/S2059798319009094 (PMC7285653; doi:10.1107/S2059798319009094)
Supplement: Supplementary file 1 [file d-75-00660-sup1.pdf]

STRUCTURAL BIOLOGY

Volume 75 (2019)

Supporting information for article:

LAT1 (SLC7A5) and CD98hc (SLC3A2) complex dynamics revealed by single-particle cryo-EM

George N. Chiduza, Rachel Johnson, Gareth S. Wright, Svetlana Antonyuk, Stephen Muench and S. Samar Hasnain

Table S1 Residues used in crosslinking experiments for the validation of LAT2 and CD98hc ectodomain docking

Residues chosen for cross-linking experiments with LAT2, their equivalent residues in LAT1. The estimated distance between these residues and residues in CD98hc ectodomain are shown as reported by Rosell *et al.*, 2014 for LAT2 and calculated from docking of the volume series of PCs 1 – 3 for LAT1 to the nearest Å.

CD98hc	LAT2	LAT1	Distance LAT2 from cross-linking (Å)	Average LAT1 distance from cryo-EM (Å)
S151	C210	G220	13.4	25 ± 2
	A315	S324	14.9	32 ± 2
S195	C210	G220	17.5	20 ± 2
	A315	S324	15.5	31 ± 2
S412	C210	G220	11.5	36 ± 2
S487	G392	S401	12.9	34 ± 1
G505	A235	V244	8.4	23 ± 1
	S441	S450	8.1	32 ± 1

Table S2 Residues predicted to be at the dimer interface of LAT2 and CD98hc and their distances from cross-linking experiments by (Rosell *et al.*, 2014).

Similarity between residues increases as similarity score goes from 1 – 9, with identical residues having no score. The Consurf score increases with decreasing variability at that position between orthologues, indicating degree of conservation.

LAT2	LAT1	Similarity score	LAT1 Consurf Score	<10 Å in LAT1 EM structure
K63	T73	7	9	No
G64	G74		9	No
E67	K77	7	1	No
F155	F161		4	No
P157	P167		6	Yes
N221	F231	6	3	Yes
F223	F233		8	Yes
L239	Y248	7	8	No
L297	L306		5	Yes
N300	E309	7	1	Yes
A301	A310		9	Yes
A303	A312		9	No
M384	I393	9	6	No
Y385	F394	9	2	No
Y390	F399	9	5	No
F393	F402		7	No
F440	V449	7	5	No
L442	F451	8	5	No
W443	W452		2	No

{"required":2,"groups":[{"required":1,"restraints":
[{"type":"residue","dmax":14.3,"dmin":
3.5,"rec_chain":"A","rec_resid":"220","lig_chain":"A","lig_resid":"1
51"},{"type":"residue","dmax":14.3,"dmin":
3.5,"rec_chain":"A","rec_resid":"324","lig_chain":"A","lig_resid":"1
51"},{"type":"residue","dmax":14.3,"dmin":
3.5,"rec_chain":"A","rec_resid":"220","lig_chain":"A","lig_resid":"1
95"},{"type":"residue","dmax":14.3,"dmin":
3.5,"rec_chain":"A","rec_resid":"324","lig_chain":"A","lig_resid":"1
95"},{"type":"residue","dmax":14.3,"dmin":
3.5,"rec_chain":"A","rec_resid":"220","lig_chain":"A","lig_resid":"4
12"},{"type":"residue","dmax":14.3,"dmin":
3.5,"rec_chain":"A","rec_resid":"401","lig_chain":"A","lig_resid":"4
87"},{"type":"residue","dmax":14.3,"dmin":
3.5,"rec_chain":"A","rec_resid":"244","lig_chain":"A","lig_resid":"5
05"},{"type":"residue","dmax":14.3,"dmin":
3.5,"rec_chain":"A","rec_resid":"450","lig_chain":"A","lig_resid":"5
05"}]},{"required":1,"restraints":[{"type":"residue","dmax":
5,"dmin":
2,"rec_chain":"A","rec_resid":"164","lig_chain":"A","lig_resid":"109
"}]}]}

Figure S1 Distance restraints provided to Cluspro for docking of CD98hc ectodomain and LAT1 in json used for ClusPro. LAT1 was submitted as receptor and CD98hc ectodomain as ligand to the server. Residues same as those given in Supplementary Table S2.

Supplementary Movie S1. Visualization of conformational dynamics of CD98hc ectodomain (tan) and LAT1 (pink), along PC1. The two subunits can be seen moving in a ratchet like motion relative to each other. The cryo-EM volumes along the principle component are shown as a mesh.

Supplementary Movie S2. Visualization of conformational dynamics of CD98hc ectodomain (tan) and LAT1 (pink), along PC2. CD98hc ectodomain and the LAT1/micelle density can be seen in a rocking motion with respect to each other. Cryo-EM volumes along the principle component are shown as a mesh.

Supplementary Movie S3. Visualization of conformational dynamics of CD98hc ectodomain (tan) and LAT1 (pink), along PC3. In this volume series, the two densities can be seen moving up and downward in opposite directions along the normal to the long axis of the larger density. Cryo-EM volumes along the principle component are shown as a mesh.
